# Elective Caesarean Delivery in a Patient With May-Hegglin Anomaly: Its Concerns and Review of the Literature

**DOI:** 10.7759/cureus.85549

**Published:** 2025-06-08

**Authors:** Yuki Julius Ng, Jin Yun Lee, Callie Foo, Wei Kang Lee

**Affiliations:** 1 Department of Anaesthesia, Sarawak General Hospital, Kuching, MYS; 2 Department of Anaesthesia, International Medical University, Kuala Lumpur, MYS; 3 Department of Paediatrics, Sarawak General Hospital, Kuching, MYS; 4 Department of Obstetrics and Gynaecology, Sarawak General Hospital, Kuching, MYS

**Keywords:** anaesthetic outcome, fetal outcome, maternal outcome, may-hegglin anomaly, perioperative outcomes

## Abstract

May-Hegglin anomaly (MHA) is an autosomal dominant haematological disorder that is commonly misdiagnosed as idiopathic thrombocytopenic purpura. The presence of leukocyte inclusion bodies differentiates it from other causes of thrombocytopenia. There is a lack of agreement on the perioperative management of patients with MHA. Our patient was a 33-year-old lady with underlying MHA, gravida 2 para 1 at 37 weeks of pregnancy, who was electively admitted for lower-segment caesarean section under general anaesthesia. The caesarean delivery was uneventful, and the mother and baby were discharged without any complications from the operation. In this patient, an intra-abdominal drain was inserted to drain any postoperative intra-abdominal bleeding; however, the drain produced minimal haemoserous drainage, which was similarly observed in her first pregnancy. We performed a systematic search of the literature on MHA and discussed the concerns of the anaesthetic, obstetric, and paediatric teams. We only found 96 pregnancies by 56 mothers with MHA and 51% (n = 49) of these pregnancies were via vaginal delivery and 43.8% (n = 42) were delivered via caesarean sections. About 85.4% (n = 82) of the 96 pregnancies had an uneventful delivery. Only one patient in the postpartum haemorrhage group was considered likely to be attributed to MHA. None of the mothers who received neuraxial anaesthesia in our literature search experienced any epidural-spinal haematoma. There were 103 infants delivered, and 85.4% (n = 88) of them had no associated complications at birth. The rest of the fetal complications were not considered to be associated with MHA. The literature suggests that mothers with MHA generally have an uneventful delivery via all methods.

## Introduction

May-Hegglin anomaly (MHA) is a rare, autosomal dominant inherited haematological disorder characterised by the presence of giant platelets, inclusion bodies in granulocytes, and thrombocytopenia [1-4]. MHA can be misdiagnosed as idiopathic thrombocytopenic purpura (ITP) and treated with ineffective treatments, such as corticosteroids and splenectomy [4]. The definitive diagnosis of MHA is limited to those with leukocytic inclusion bodies, due to aggregates of abnormal non-muscle myosin heavy chain and no other organ dysfunction [2]. The presence of these inclusion bodies in leukocytes but not platelets differentiates MHA from other causes of thrombocytopenia, such as ITP [2]. Despite its recognition as a genetic disorder associated with a specific gene mutation, there is still a lack of consensus on the optimal perioperative management for patients with MHA undergoing surgery. We describe a patient diagnosed with MHA who underwent an elective lower-segment caesarean section under general anaesthesia with prophylactic platelet transfusion and tranexamic acid before induction. We also performed a systematic search and review of the literature to discuss clinical aspects of the findings in terms of obstetric, anaesthesia, and paediatric concerns. This case was written according to the CARE (Case Report) guidelines.

## Case presentation

We report a case of a 33-year-old pregnant woman, gravida 2 para 1, at 37 weeks of amenorrhea, electively admitted to our hospital for lower-segment caesarean section, in view of an underlying MHA. Antenatally, she had a previous history of caesarean section done for her first pregnancy; her postoperative outcome was uneventful, with an intraoperative drain showing minimal drainage with an estimated blood loss of 500 ml intraoperatively. Throughout both of her pregnancies, she did not have any antenatal or antepartum haemorrhage. Before admission, she did not have any spontaneous bleeding, easy bruising, petechiae, gum, nasal, or mucosal bleeds, or any previous requirement for transfusion. Her bleeding stopped well (compared with her peers) in the past from minor trauma and injury. She was otherwise not in labour, and had irregular tightening and good fetal movement. Her cervix was closed, and she had zero contractions in 10 minutes. Her bedside antenatal scans were corresponding to date, and the cardiotocograph was reassuring.

A multidisciplinary team meeting between obstetrics, haematology, anaesthesiology, and paediatrics was carried out to keep each team updated and ready for delivery. The obstetrics team led the discussion and brought the other team together to discuss the best method of delivery and a proper plan with anticipation. The pathophysiology and the bleeding risk were explained and stratified by the haematology team and further discussed with anaesthesiology to provide the lowest risk possible. The paediatrics team were informed of the method of delivery and its potential risk to the newborn child.

Delivery outcome

The patient underwent a lower-segment caesarean section under general anaesthesia. Intraoperatively, four units of platelets and 1 g of tranexamic acid were given before skin incision. The operation was uneventful, with an estimated blood loss of 500 ml. The baby was born cephalic, with clear liquor. An intra-abdominal drain was inserted. Postoperative vaginal examination showed no blood clots upon evacuation, and there were no bruises seen over the postoperative site. The lochia loss was reduced on day two postoperatively. The intra-abdominal drain produced minimal haemoserous content and was taken out after two days postoperatively (this was also previously performed for her first pregnancy).

Fetal outcome

Her baby boy was born with an Apgar score of 5 at one minute, 9 at five minutes, and 10 at 10 minutes. Post delivery, her baby required two cycles of positive pressure ventilation and was admitted to the nursery for close monitoring. Birth weight was 2.84 kg. After admission, the baby was able to oxygenate; however, he remained in the nursery for phototherapy for neonatal jaundice secondary to G6PD. The baby’s platelet count was 345k/uL.

**Figure 1:**
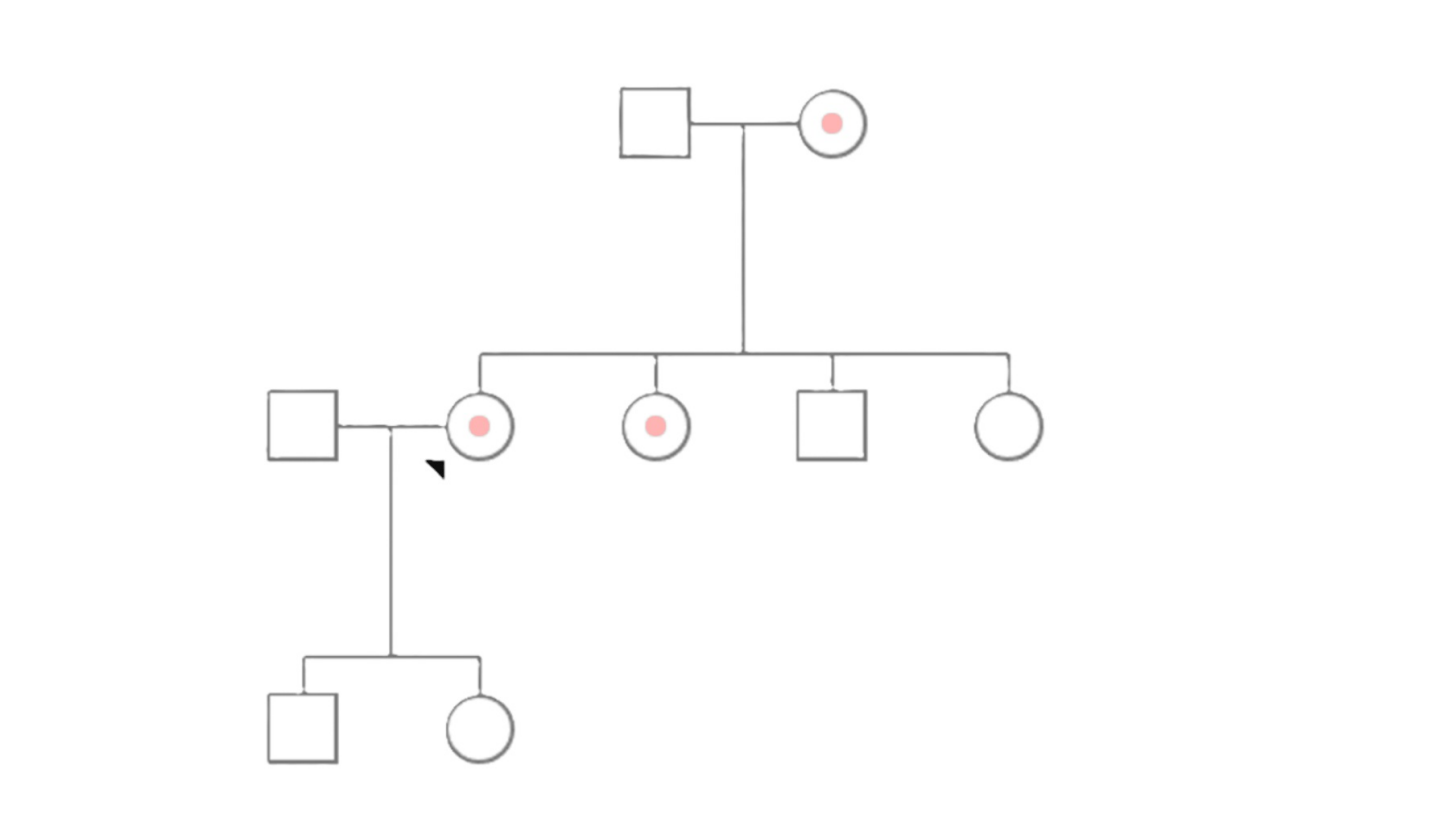
Pedigree of the patient’s family who is affected by MHA. Arrowhead: our patient. Dotted figure: May-Hegglin anomaly (MHA).

Her previous baby girl was born flat, requiring intubation, likely due to the general anaesthesia. The baby girl is currently well and healthy; however, the patient reported that the doctors mentioned the baby girl has low platelets and is likely to have MHA. After her first pregnancy, her thrombocytopenia was investigated, and since then her family has been screened for MHA (Figure 1). Blood investigations of the patient are presented in Table 1.

**Table 1:** The patient's blood parameters on admission.

Blood parameters	Result	References
Haemoglobin	164	130 - 170 g/L
White cells	6.9	4.0 - 11.0 x10^9/L
Platelets	56	150 - 400 x10^9/L
Prothrombin time	9.8	10 - 13.2 s
International normalised ratio	0.87	0.8 - 1.2
Activated partial thromboplastin time	23.8	25.1 - 36.5 s
Total bilirubin	5	<21 umol/L
Alkaline phosphatase	32	30 - 130 U/L
Alanine aminotransferase	40	10 - 60 U/L
Sodium	138	133 - 146 mmol/L
Potassium	4.2	3.5 - 5.3 mmol/L
Urea	3	2.5 - 7.8 mmol/L
Creatinine	60	59 - 104 umol/L
Estimated glomerular filtration rate (eGFR)	>90	>90 ml/min

Her prothrombin time was 9.8 seconds, international normalised ratio was 0.87, and activated partial thromboplastin time was 23.8 seconds. Her platelet trend was as follows: 63k/uL - 58k/uL - four units of platelets transfusion - 56k/uL - 90k/uL (postoperation) - 81k/uL. Her previous preoperative platelet trend was as follows: 44k/uL - 57k/uL (postoperation) - 52k/uL.

Peripheral blood film (PBF) showed evidence of low platelets at 47k/uL during her first pregnancy. There was the presence of basophilic cytoplasmic inclusion bodies seen in neutrophils resembling Döhle bodies. Her platelets were mildly reduced (26-33/HPF), with no platelet clumps or fibrin seen, but the presence of large and giant platelets was noted, which were suggestive of MHA.

Follow-up

She was under haematology and obstetrics clinic follow-up. In both visits, the patient reported no signs of haemorrhage during and after puerperium and did not require any intervention. However, she underwent close monitoring under the haematology team every month for the past six months to catch any bleeding tendencies from the operation. The patient had an otherwise uneventful pregnancy.

## Discussion

**Figure 2:**
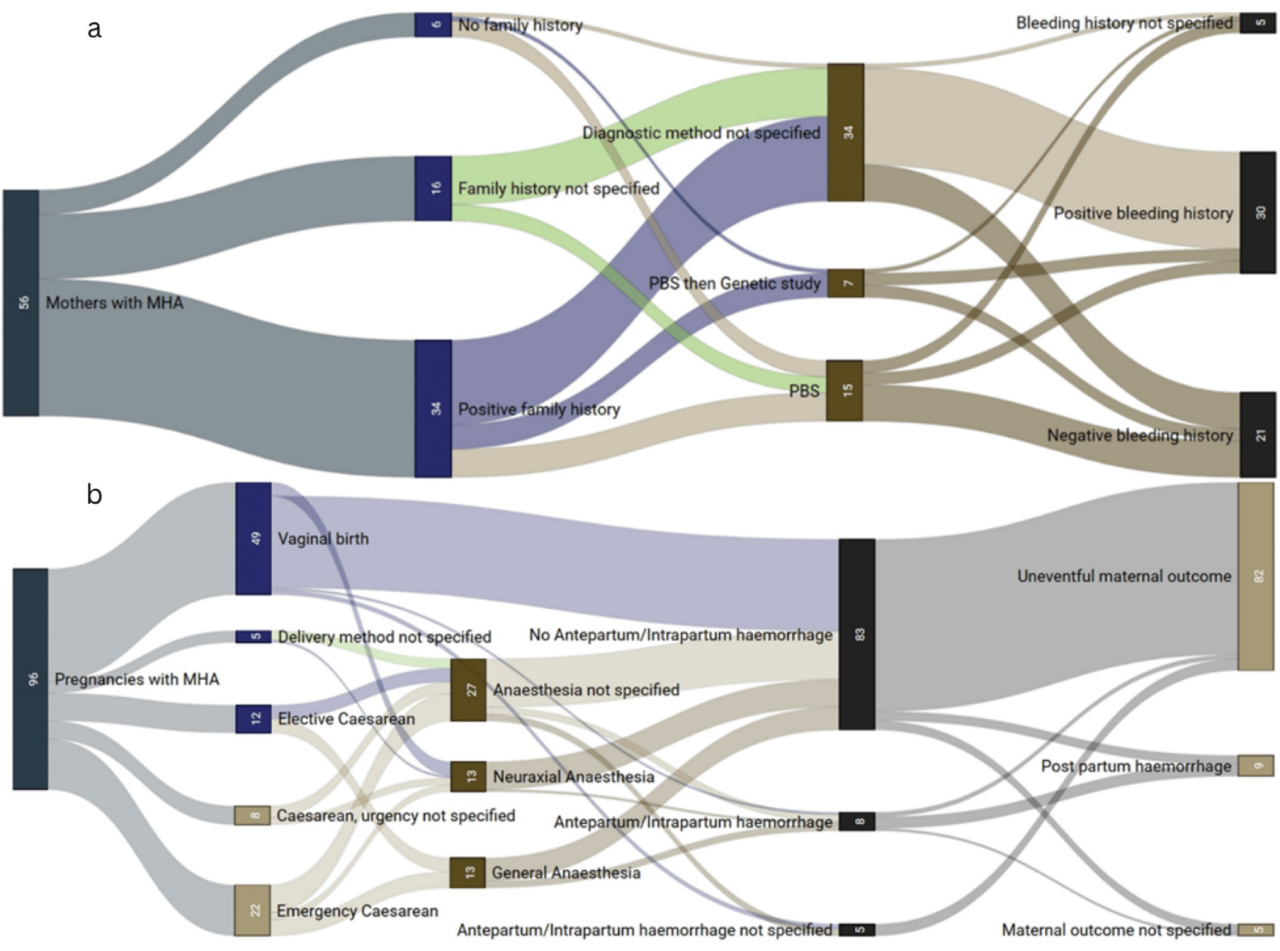
Panel a: Proportion of mothers with family history, methods of diagnosis of MHA, and their previous history. Panel b: Proportion of number of pregnancies, mode of delivery, anaesthesia, and maternal outcomes. MHA: May-Hegglin anomaly; PBS: peripheral blood smear.

MHA is an autosomal dominant giant platelet disorder characterised by abnormally large platelets with defective leucocytes and thrombocytopenia [3]. A literature search was conducted using PubMed, MEDLINE, Embase, CENTRAL, Global Health, Global Index Medicus, Scopus, Canadian Agency for Drugs and Technologies in Health (CADTH), Networked Digital Library of Theses and Dissertations (NDLTD), Public Affairs Information Service (PAIS), bioRxiv, Google Scholar, and Google search. Our search strategy was “May Hegglin anomaly” AND “pregnancy”. We included all languages, case reports, case series, and cohort studies that included primary data analysis. Articles in languages other than English were translated to English with a visual multilingual neural machine tool for further interpretation and data extraction. We utilised articles that analysed secondary data to further our search for published literature. A total of 100 articles were found after removing repeated titles, but only 40 were selected. Clinical data were extracted from these articles, which concern obstetrics, anaesthesia, and paediatric concerns where possible. There were 96 reported pregnancies by 56 mothers, which are collated and summarised (Figure 2, panel a).

The preferred method of diagnosis for MHA is a genetic study for the MYH9 gene, present on chromosome 22q12-2A, due to its rarity; however, a thorough evaluation with peripheral blood smear (PBS) is recommended in those with a positive family history of bleeding diathesis. Among the 56 mothers, 26.8% (n = 15) were diagnosed with PBS, 60.7% (n = 34) were unspecified, and 12.5% (n = 7) were diagnosed with PBS and confirmed with genetic studies. Among 56 mothers, 60.7% (n = 34) had a positive family history, 10.7% (n = 6) had a negative family history, and 28.6% (n = 15) had an unspecified family history. The majority of mothers (53.6%, n = 30) had a positive bleeding history, 37.5% (n = 21) did not have a bleeding history, and 8.9% (n = 6) of the reported mothers did not specify a bleeding history. Curiously, 60% (n = 9) of those who were diagnosed with PBS had a negative bleeding history. However, 80% (n = 24) of those who had positive bleeds did not specify the diagnostic method of MHA. But for those who were confirmed with PBS, 20% (n = 3) had a history of bleeding, which is still a considerably high proportion from the pooled population. Of those who had a positive family history of MHA, 61.8% (n = 21) of mothers were not reported with the method of diagnosis. The common misdiagnosis is ITP due to its similarities in clinical presentation; hence, genetic testing should be done to confirm the diagnosis.

In our literature review (Table 2), there were a total of 96 pregnancies and the majority (51%, n = 49) were via vaginal delivery, followed closely by lower-segment caesarean section (LSCS) at 43.8% (n = 42), and 5.2% (n = 5) of deliveries were unspecified. Of those pregnancies that were delivered via caesarean section, 52.4% (n = 22) were emergency deliveries, 28.6% (n = 12) were elective, and 11.9% (n = 5) were unspecified. A total of 64.3% (n = 27) of those who required caesarean section did not report the mode of anaesthesia, 31% (n = 13) were done under general anaesthesia, and 14.3% (n = 6) were done under neuraxial anaesthesia. Epidural analgesia was given to three mothers who gave birth via six vaginal deliveries. The majority of pregnancies (86.5%, n = 83) did not have any antepartum or intrapartum haemorrhage, only 8.3% (n = 8) experienced either antepartum or intrapartum haemorrhage, and the rest were not specified. Among those who did not have a history of antepartum or intrapartum haemorrhage, only 4.8% (n = 4) resulted in postpartum haemorrhage, and the other five pregnancies that had postpartum haemorrhage were from those mothers who had a history of antepartum or intrapartum bleeding. Postnatally, the majority of mothers had an uneventful recovery at 85.4% (n = 82) whilst only 9.7% (n = 9) developed postpartum haemorrhage. Four of them were treated with blood products, and 5.2% (n = 5) were unspecified. Among the nine mothers with postpartum haemorrhage, we only attributed one patient with bleeding potentially from MHA from a surgical site oozing. The rest had other risk factors such as placenta praevia (n = 1), HELLP (haemolysis, elevated liver enzymes, and low platelets) syndrome (n = 1), uterine atony (n = 2), and unspecified(n = 4). The descriptive data of this review suggest a possible reassurance of uneventful pregnancies; however, those with a history of bleeding have a potentially increased risk of postpartum haemorrhage. This suggests that individuals affected by MHA may have an increased risk of bleeding, which was seen in other cases [4]. To properly stratify the risk of bleeding and its clinical implications, more studies with higher levels of evidence and proper data collection should be performed. Given the rarity of this disease, it would be more feasible for a multicentre prospective study to observe risks and possible guidelines to guide future clinicians further (Figure 2, panel b).

**Table 2:** Review of the literature and the collated data of pregnant mothers with MHA. FBC: full blood count; SVD: spontaneous vertex delivery; ITP: immune thrombocytopenic purpura; AUB: abnormal uterine bleeding; MHA: May-Hegglin anomaly; GA: general anaesthesia; SA: spinal anaesthesia; LSCS: lower segment caesarean section; IUGR: intrauterine growth restriction; IOL: induction of labour; PPH: postpartum haemorrhage; IVIG: intravenous immunoglobulin; PBS: peripheral blood smear; MRI: magnetic resonance imaging; CSF: cerebrospinal fluid; PROM: preterm rupture of membrane; PT: prothrombin time; aPTT: activated partial thromboplastin time; INR: international normalised ratio; URTI: upper respiratory tract infection; HELLP: haemolysis, elevated liver enzymes, and low platelets; ELLSCS: elective lower-segment caesarean section; ISTH: International Society on Thrombosis and Haemostasis; P/w: presented with; ROM: rupture of membrane; SROM: spontaneous rupture of membrane; MCDA: monochorionic diamniotic; CVI: cavum veli interpositi; FH: family history; APH: antepartum haemorrhage; O2: oxygen; FM: face mask; IVI: intravenous infusion; HLA: human leukocyte antigen; IUD: intrauterine device; TXA: tranexamic acid; DDAVP: 1-deamino-8-d-arginine vasopressin (desmopressin).

Author, year published	Number of pregnancies in the paper	Background	Country (paper published)	Ethnicity	Age	Family history of MHA	G/P/M (M = miscarriage)	Methods of diagnosis	Time of diagnosis (e.g., 10 gestational weeks)	Age of diagnosis with MHA	Bleeding history prior to pregnancy	Management plan	Mode of delivery	If operated, the mode of anaesthesia	Platelets prior to delivery	PT	aPTT	INR	Bleeding time	Platelet transfusion	Bleeding complication (maternal)	Other complications (maternal)	Estimated blood loss (ml)	Fetal complications	Fetal outcome	Maternal outcome
Takashima et al. (1992) [5]	1	No history of bleeding, underwent appendicectomy at 15 and was uneventful. Diagnosed as ITP at 23 after p/w URTI and treatment with 50 mg prednisone/day	Japan	Japanese	26	Unknown	Primigravidarum	PBS	17+2 weeks of gestation	26	No bleeding tendency	None	SVD	-	35,000	Not specified	Not specified	Not specified	Not specified	No	No	Uneventful	Not specified	Uneventful, MHA + in utero following cordocentesis	Uneventful	Uneventful
Muzannar et al. (2017) [6]	3	Initially diagnosed with ITP, treated with steroids, multiple platelet transfusions, and 4 cycles of rituximab. Initially, ITP was unresponsive to treatment, and splenectomy was done with minimal blood loss	Saudi Arabia	Not specified	Not specified	Yes	G5P3M2	PBS, bone marrow biopsy	After 2nd pregnancy	Not specified	No bleeding tendency	1: platelet 18 units and cryo 6 units + IVIG. 2: 6 units of platelets, IVIG, and prednisolone. 3: 6 units of platelets	1: LSCS. 2: SVD. 3: LSCS	1: GA. 3: Epidural	1:22,000. 2: 7,000. 3: 13,000	Normal	Normal	Normal	Normal	Yes	1: No. 3: Not specified	Uneventful	Not specified	Not specified	Not specified	Uneventful
Magann et al. (1999) [7]	1	Initially misdiagnosed as ITP, treated with steroids and IVIG	United States of America	Not specified	28	Unknown	Primigravidarum	PBS	Not specified	28	No bleeding tendency	6 units of platelets	LSCS	GA	29,000	Not specified	Not specified	Not specified	Not specified	Yes	No	Uneventful	Not specified	Ambiguous genitalia, fetal growth restriction, MHA	Uneventful	Uneventful
Guruparan et al. (2021) [3]	1	-	Sri Lanka	Sri Lankan	29	Yes	Primigravidarum	PBS	Not specified	24, following family screening	No bleeding tendency	6 units of platelets prior to operation	LSCS	SA	30,000	Not specified	Not specified	Not specified	Not specified	Yes	No	Uneventful	Not specified	Uneventful	Uneventful	Uneventful
Nelson et al. (1993) [8]	2	-	United States	Caucasian	31	Unknown	1: G3P0M2. 2: G4P1M2	Not specified	Prior 3rd pregnancy	Not known	History of 2 miscarriage during the 1st trimester of 1st and 2nd pregnancy	No medication was given to the mother. The patient underwent elective LSCS under GA in view of the risk of fetal thrombocytopenia	1: LSCS. 2: LSCS	1: GA. 2: GA	1: 84,000. 2: 80,000	Not specified	Not specified	Not specified	Not specified	No	No	Uneventful	1: 600. 2: Not specified	1: MHA (+). 2: Uneventful	Uneventful	Uneventful
Kotelko (1989) [9]	1	P/w at 41 weeks with ROM. Observed for 24 hours, and a trial of labour was offered with induction of labour and augmentation, but failed.	United States of America	Not specified	31	Unknown	Primigravidarum	Not specified	Prior to pregnancy	Not Known	No bleeding tendency, even during tooth extraction	6 units of type-specific platelets, 100% O2 FM, IVI 1500 ml lactated Ringer's, spinal anaesthesia with 5% lidocaine with 1:200,000 epinephrine & 0.5 mg morphine sulphate. Emergent LSCS due to failed IOL	LSCS	SA	24,000	Normal	Normal	Normal	Normal	No	Yes	Uneventful	700	MHA (+)	Uneventful, male, alive. platelet count of 41,000	PPH secondary vaginal tear
Fishman et al. (2009) [10]	7 (3 mothers)	Incidental finding on a blood test on one of the family members. 3 sisters with MHA on anaesthesia during delivery	United States of America	Not specified	Not specified	Yes	Para 3	Not specified	Prior to pregnancy	Not specified	No bleeding tendency	Not specified	1: SVD. 2: SVD. 3: SVD	1: Epidural. 2: Epidural. 3: Epidural	1: 100,000. 2: 47,000. 3: 26,000	Normal	Normal	Normal	Normal	No	No	Uneventful	Not specified	Not specified	Uneventful, MHA+, one neonate had 35 platelet count	Uneventful
			United States of America	Not specified	Not specified	Yes	Para 2	Not specified	Prior to pregnancy	Not specified	No bleeding tendency	Not specified	1: SVD. 2: SVD	1: Epidural. 2: Epidural	1: 48,000. 2: 26,000	Normal	Normal	Normal	Normal	No	No	Uneventful	Not specified	Not specified		Uneventful
			United States of America	Not specified	Not specified	Yes	Para 2	Not specified	Prior to pregnancy	Not specified	No bleeding tendency	Not specified	1: LSCS. 2: LSCS	1: SA. 2: SA	1: 14,000. 2: 81,000	Normal	Normal	Normal	Normal	No	No	Uneventful	Not specified	Not specified		Uneventful
Amodeo et al. (2021) [11]	Twin pregnancy	Monochorionic, diamniotic twins	Italy	Not specified	32	Unknown	Primigravidarum	PBS	Postpartum day 1	32	No bleeding tendency	Emergent LSCS at 32+1 weeks due to altered umbilical and medial cerebral artery flowmetry of the second fetus	LSCS	Not specified	Not specified	Not specified	Not specified	Not specified	Not specified	No	No	IUGR of twin 1 and cerebral anomalies of both twins on ultrasound. Fetal MRI showed mild ventriculomegaly associated with a cyst in the velum interpositum	Not specified	Both twins are MHA (+) with mild ventriculomegaly and a cyst of the velum interpositum without evidence of obstruction of CSF circulation	Twin 1: Male, alive. Weight: 1490g. Platelet: 12,000. No bleeding complications. Twin 2: Male, alive. Weight: 2010 g. Platelet: 24,000. No bleeding complications	Uneventful
Landy et al. (1987) [12]	2	1st and 3rd pregnancy. 3rd pregnancy with antiplatelet antibody positive and placenta previa	United States of America	Caucasian	17 (1st), 24 (3rd)	Yes	1: Primigravida. 3: G3P1M1	Not specified	After 1st pregnancy	18	No known bleeding history, bleeding after cervical cone biopsy	Not known (1st), HLA-matched platelet, but did not correct bleeding time, cryoprecipitate and steroids were also given. Emergent LSCS in view of placenta previa (3rd)	1: SVD. 3: LSCS	3: Not specified	3: 22,000	Not specified	Not specified	Not specified	Not specified	1: No. 2: Cryoprecipitate. 3: No	1: No. 2: No. 3: Heavy painless vaginal bleed postpartum	-	1: No. 3: 1000 ml	1. Uneventful. 3: MHA (+)	Uneventful	1: PPH, transfused 8 pints of packed cells. 3: PPH, transfused platelets and cryoprecipitate
Pajor et al. (1999) [13]	1	Chronic proliferative glomerulonephritis, initially misdiagnosed with ITP and treated with steroids and splenectomy	Hungary	Hungarian	25	Unknown	Para 2	PBS	10 years postpartum	25	Easy bruising prior to first pregnancy	1: platelet concentrate. 2: not specified	1: LSCS. 2: LSCS	Not specified	1: 70,000. 2: 100,000	Not specified	Not specified	Not specified	Not specified	1: Yes. 2: Not specified	No	Uneventful	Not specified	Uneventful	Uneventful	Uneventful
Takabayashi et al. (2007) [14]	1	Antiplatelet antibody (-)	Japan	Japanese	26	Unknown	Primigravidarum	Not specified	Not known	Not known	No bleeding tendency	Elective LSCS under GA	LSCS	GA	49,000	Not specified	Not specified	Not specified	Not specified	No	No	No	Not known	Not specified	Uneventful	Uneventful
Binder et al. (2003) [15]	2	Initially misdiagnosed as ITP (minimal response to steroids); uneventful pregnancy course	Prague	Not specified	Not specified	Unknown	Not specified	Not specified	Routine antenatal blood test (wrong diagnosis)	Not known	Not specified	Corticosteroid administered, response was minimal	LSCS	GA	34,000	Not specified	Not specified	Not specified	Not specified	Yes	No	No	400 ml	Uneventful	Uneventful	Uneventful
		Initially misdiagnosed as ITP (minimal response to steroids ), developed signs of pre-eclampsia	Prague	Not specified	Not specified	Unknown	Not specified	Not specified	Routine antenatal blood test (wrong diagnosis)	Not known	Not specified	Trial of labour with induction of labour (unsuccessful). Subsequent LSCS. Corticosteroid administered, response was minimal	LSCS	GA	27,000	Not specified	Not specified	Not specified	Not specified	Yes	No	No	700 ml	Uneventful	Uneventful	Uneventful
Kim et al. (2012) [16]	1	No known medical illness	Korea	Korean	39	Yes	Primigravidarum	PBS, bone marrow biopsy, genetic study (MYH9)	12 weeks of gestation	39	Not specified	Not specified	LSCS	Not specified	Not specified	Normal	Normal	Normal	Normal	Not specified	Not specified	Not specified	Not specified	Bilateral temporal cephalohematoma, MHA (+)	Uneventful	Uneventful
Urato et al. (1998) [17]	1	P/w SROM and contraction	United States of America	Not specified	31	Yes	Primigravidarum	FBC	Prior pregnancy	29	No bleeding tendency, slight heavy periods for 4-5 days	Not specified	SVD	-	16,000	Not specified	Not specified	Not specified	Not specified	No	No	No	Not specified	Uneventful	Uneventful	Uneventful
Chatwani et al. (1992) [18]	1	P/w spontaneous ROM at >39 weeks. Ultrasound showed no frank breech presentation of the fetus	United States of America	Caucasian	28	Unknown	G4P0M3	Not specified	Prior pregnancy	Several years prior	Bleeding gums 1 week prior to admission. Otherwise, no bleeding tendency	12 units of platelet transfusion prior to surgery	LSCS	Not specified	34,000	Not specified	Not specified	Not specified	Not specified	Yes	No	No	Not specified	MHA (-)	Uneventful	Uneventful
Chabane et al. (2001) [19]	3	Developed PROM with fever and abnormal labour progress	France	French	24	Unknown	Para 3	FBC/PBS	24 weeks of gestation	24	No bleeding tendency	Platelet transfusion for the first pregnancy	1: LSCS. 2: LSCS. 3: LSCS	Not specified	1: 45,000. 2: Not specified. 3: 32,000	Normal	Normal	Normal	Normal	1: Yes. 2: No. 3: No	No	No	Not specified	1: MHA (-). 2: MHA (-). 3: MHA (+)	Uneventful	Uneventful
Siddiqui et al. (1991) [20]	1	Antiplatelet antibody (+); initially misdiagnosed as immune thrombocytopenia. Subsequently, not responsive to steroids, IVIG, and splenectomy	United States	Caucasian	22	Yes	Primigravidarum	Not specified	After the current pregnancy	Not known	No bleeding tendency	Emergent LSCS due to HELLP syndrome	LSCS	GA	22,000	Not specified	Not specified	Not specified	Not specified	No	Yes, intermittent vaginal bleeding	HELLP Syndrome	Not known	MHA (-)	Uneventful	Persistent thrombocytopenia despite plasmapheresis and continued steroids post delivery
Ishii et al. (1993) [21]	1	-	Japan	Japanese	25	Yes	Primigravidarum	Routine antenatal blood test	During routine antenatal blood test follow-up	25	Not specified	Elective LSCS under GA due to the risk of fetal thrombocytopenia	LSCS	GA	49,000	Not specified	Not specified	Not specified	Not specified	Yes	No	No	997	MHA (-)	Uneventful	Uneventful
Kinsella et al. (1999) [22]	1	Initially misdiagnosed as ITP (steroid and IVIG used for autoimmune thrombocytopenia). Antiplatelet antibody (-)	United States	Not specified	28	Yes	Primigravidarum	Routine antenatal blood test	Not known	28	No bleeding tendency	Elective LSCS due to the risk of fetal thrombocytopenia	LSCS	GA	29,000	Not specified	Not specified	Not specified	Not specified	Yes	Not specified	No	Not known	Intrauterine growth restriction with ambiguous genitalia. MHA (+)	Uneventful	Uneventful
Duff et al. (1985) [23]	1	The patient had concurrent rhesus sensitisation	USA	Not specified	23	Yes	Primigravidarum	Not specified	Prior to the current pregnancy	19	Bleeding after tooth extraction	Platelet transfusion x2 and blood transfusion postoperation due to prolonged bleeding time. Elective LSCS due to uncertain risks of vaginal delivery and patient's choice	LSCS	SA	67,000	Not specified	Not specified	Not specified	Not specified	Yes	No	none	1000	MHA (+)	Uneventful	Oozing surgical site, transfused platelets
Fayyad et al. (2002) [24]	3	Incidental finding of low platelet count (40) during 1st trimester of her first pregnancy. Platelet count ranged from 40 to 61 throughout her pregnancy	United Kingdom	Not specified	25	Unknown	Primigravidarum	PBS	During 1st trimester of her first pregnancy	25	No bleeding tendency	1: Emergency LSCS at 36 weeks because of sudden severe proteinuric hypertension along with elevated liver enzymes. 2: Sudden cessation of fetal movements. Subsequent u/s showed IUD. 3: Patient had two-week assessments of fetal growth and weekly biophysical profile by ultrasound from 24 weeks onwards. Results were normal. Elective LSCS planned at 37 weeks	1: LSCS. 2: SVD. 3. LSCS	Not stated	1: 50,000. 2: 162,000. 3: 129,000	Not specified	Not specified	Not specified	Not specified	No	No	None	1: Average blood loss. 2. Not specified. 3: Average blood loss	1: No complications. 2: Stillbirth (no thrombocytopenia). 3: No complications	1: Uneventful. 2: Intrauterine fetal demise, macerated, female, weight: 3.4 kg. Placental changes suggest uteroplacental insufficiency caused fetal demise. 3: Uneventful	Uneventful
Tordjeman et al. (1996) [25]	1	Diagnosed with MHA after a check-up due to an episode of acute abdominal pain	France	Not specified	30	Unknown	Primigravidarum	Not specified	Prior to pregnancy	21	Yes, easy bruising	Forceps vaginal delivery due to failure to progress	Forceps delivery	-	54,000	Not specified	Not specified	Not specified	Not specified	No	No	None	Not known	MHA (-)	Uneventful	Uneventful
Garcia-Horton et al. (2020) [26]	1	-	Canada	Not specified	29	Yes	Not specified	PBS, genetic study	8 weeks of gestation	29	No bleeding tendency	Yes	SVD	Epidural	32,000	Not specified	Not specified	Not specified	Not specified	Yes	No	No	Not known	MHA (-)	Uneventful	Uneventful
Favier et al. (2018) [27]	2	Underwent appendectomy at 15 and received a platelet transfusion, but developed angioedema and anaphylaxis. Had p.Met1934 TrpfsX mutation in exon 40 of the MYH9 gene. ISTH bleeding score was 3	France	Not specified	41	Unknown	Para 2	Genetic study	Infancy	Infancy	Yes (epistaxis, minor wounds, and cutaneous)	1: No special management. 2: eltrombopag 50 mg/day at 36 weeks of gestation and platelets at 30	1: LSCS. 2: LSCS	1: GA. 2: SA	1: 26,000. 2: 179,000	Not specified	Not specified	Not specified	Not specified	No	1: Uterine atony treated with sulprostone. 2: bleeding from the incision site after starting LMWH 12 hours after LSCS. No transfusion. Hb11.2 post surgery	no	Not specified	1: MHA (+). 2: MHA (+)	Uneventful	Uneventful
Giordano et al. (2020) [28]	1	Had deafness, macrothrombocytopenia, and bleeding episodes since childhood, MCDA twin pregnancy with CVI cyst diagnosed at 19 weeks of gestation via USG	Italy	Italian	32	Unknown	Not specified	Genetic study	Prior pregnancy	30	Yes (epistaxis and menorrhagia)	IV TXA 1 g given prior to GA	LSCS	GA	16,000	Not specified	Not specified	Not specified	Not specified	Yes	No	No	Not known	Twin 1: MHA (+), CVI cyst. Twin 2: MHA (+), CVI cyst	Uneventful	Uneventful
Conte et al. (2018) [29]	1	Initially misdiagnosed as ITP during the first pregnancy, unresponsive to steroids and gamma globulin	Chile	Not specified	51	Yes	Para 2	PBS, bone marrow biopsy, genetic study	Age 50	50	No bleeding tendency	Not specified	1: SVD. 2: Not specified	Not specified	-	Normal	Normal	Normal	Normal	Not specified	Not specified	Uneventful	Not specified	Not specified	Not specified	Uneventful
Chin et al. (2017) [30]	1	Diagnosed with ITP at 5 years old; however, unresponsive to steroids with subsequent loss to follow-up. Peripheral blood smear at 24 weeks of gestation showed signs of MHA	Malaysia	Not specified	26	Yes, only known after a genetic study was done for the patient's family tree	Not specified	Genetic study	After the current pregnancy	26	No bleeding tendency	Spontaneous labour at 38 weeks with a platelet count of less than 10. Given platelet transfusion (unknown units)	SVD	-	<10,000	Not specified	Not specified	Not specified	Not specified	Yes	No	None	200	Uneventful	Uneventful	Uneventful
Rosen et al. (1997) [31]	1	She and her mother had AUB. 303 mg/dl --> 458 mg/dl fibrinogen level (N: 180-350)	Germany	German	25	No, but the mother was symptomatic	Primigravidarum	PBS, aggregometer	11 weeks + 4 days	25	Yes, prolonged menstrual bleeding	ELLSCS at 38 + 4 weeks for breech and given DDAVP 8 amp postoperation	LSCS	Not stated	26,000	117% -> 129%	Not mentioned	Not mentioned	More than 20 minutes	No	No	No	Not mentioned	Uneventful	Uneventful	Managed with 8 amp of DDAVP IV
Yamashita et al. (2016) [32]	1	The patient had a low platelet count, and a subsequent peripheral blood smear in the 1st trimester demonstrated suspicion of MYH9 disorder. Pure tone audiometry showed bilateral sensorineural hearing loss, and she had underlying gestational diabetes mellitus treated with medico-nutritional therapy	Japan	Japanese	35	FH (mother and grandmother) of thrombocytopenia and difficulty hearing	Primigravidarum	PBS	After delivery	35	No bleeding tendency	Elective LSCS at 37 weeks in view of breech presentation	LSCS	GA	60,000	Not specified	Not specified	Not specified	Not specified	No	No	None	867 ml	MHA (-)	Uneventful	Uneventful
Safiullina et al. (2022) [33]	3	Menarche at 14, first pregnancy at the age of 34	Russia	Russian	34	Yes	Not specified	PBS, genetic study	Prior pregnancy, there was an incidental finding of platelets at 50	29	Yes, AUB, epistaxis, ecchymosis, and easy bruising. No episodes of bleeding/APH	ELLSCS at 38 weeks	LSCS	Not specified	Not specified	Not specified	Not specified	Not specified	Not specified	Yes	No	None	Not mentioned	MHA (+)	Uneventful	Uneventful
		Menarche at 14, first noted bleeding tendencies at age 24 (petechiae and platelet count at 28). Treated with oral steroids, but no effect, so splenectomy was done with no effective outcomes	Russia	Russian	36	Yes	Not specified	PBS, genetic study	After pregnancy	38	Yes, petechiae at the lower extremities	ELLSCS at 28 weeks	LSCS	Not specified	Not specified	Not specified	Not specified	Not specified	Not specified	Ambiguous	Yes	None	Not mentioned	Uneventful	Uneventful	Uneventful
		Born in 1959, menarche at age 14	Russia	Russian	Not specified	Yes	Para 2	Not specified	After pregnancy	57	Yes, heavy menstrual bleeding	Not specified	Not specified	Not specified	Not specified	Not specified	Not specified	Not specified	Not specified	No	No	No	Not mentioned	Uneventful	1: Uneventful. 2: Uneventful	Uneventful
García Vallejo et al. (2014) [34]	1	-	Mexico	Latina	28	No	Primigravidarum	PBS	During 3rd trimester	Discovered at 18 but confirmed at 28	No bleeding tendency	Emergency LSCS at 39+5 weeks following failed IOL with prostin. Pain management: IV paracetamol 1 g every 6 hours & IV pethidine 25 mg every 4 hours alternating	LSCS	GA	20,900	Normal	Normal	Normal	Normal	No	No	No	500 ml	Uneventful	Uneventful	Uneventful
Kumazawa et al. (2019) [35]	1	Diagnosed with ITP at age 4, spontaneous conception and platelet count was 4x10^4 at 13+2 weeks	Japan	Japanese	29	Yes, family history of thrombocytopenia	Primigravidarum	PBS	13+2 weeks	29	No bleeding tendency	IOL at 38+2 due to oligohydramnios (4.9 cm)	SVD	-	134,000	Not specified	26.6 s	1	Not specified	No	No	No	275 ml	MHA (+)	Uneventful	Uneventful
Gausis et al. (1969) [36]	2	During week 28 of 2nd pregnancy, had vaginal bleeding and epistaxis	Italy	Caucasian	32	No	1: primigravida. 2: G2P1	PBS	During week 28 of 2nd pregnancy	36	Easy bruising, vaginal bleeding, epistaxis	1: Not known. 2: Spontaneous vaginal delivery at 28 weeks	1: Not specified. 2: SVD	Not specified	1: 50,000. 2: 50,000	Not specified	Not specified	Not specified	Not specified	No	1: Frequent epistaxis. 2: Vaginal bleeding and extensive leg bruises	No	Not known	Uneventful	1: Uneventful. 2: Stillborn, macerated dead baby	Uneventful
Budde et al. (1979) [37]	1	Hematoma after breast operation	Germany	Not specified	21	Unknown	Primigravidarum	Not specified	Prior to the current pregnancy	Not known	Postoperative bleeding	Not specified	SVD	-	30,000	Not specified	Not specified	Not specified	Not specified	No	No	No	Average	Uneventful	Uneventful	Uneventful
Bettaieb et al. (1991) [38]	2	1: Negative antiplatelet antibody. 2: Positive antiplatelet antibody, history of prior twice miscarriages	France	Not specified	Not specified	Yes	1: G1P0M2. 2: G2P1M2	Not specified	Prior to 1st pregnancy (1st week of life)	Not known	Epistaxis, menorrhagia, easy bruising, twice miscarriages	1: 3 times platelet transfusion. 2: 3 times platelet transfusion and IVIG used to prevent neonatal alloimmune thrombocytopenia. Elective LSCS due to the risk of fetal thrombocytopenia	1: LSCS. 2: LSCS	Not specified	1: 10,000. 2: Not specified	Not specified	Not specified	Not specified	Not specified	No	No	History of prior twice miscarriages	Not known	1: Uneventful. 2: MHA (+)	1: Uneventful. 2: Uneventful	Uneventful
Turnquest et al. (1994) [39]	2	Initially misdiagnosed as ITP and thrombocytopenia unresponsive to steroids, IVIG, and splenectomy	United States	Not specified	19	Yes	Primigravidarum	Routine antenatal blood test	During the antenatal check-up in 1st pregnancy	19	Gum and nose bleeding	1: Cordocentesis at 38 weeks. 2: Cordocentesis at 37 weeks	1: LSCS. 2: LSCS	Not specified	1: 82,000. 2: 37,000	Not specified	Not specified	Not specified	Not specified	Not specified	No	1: Non-reassuring fetal tracing. 2: Footling breech presentation after spontaneous rupture of membranes	Not known	1: Uneventful. 2: Uneventful	1: Uneventful. 2: Uneventful	Uneventful
Kumi et al. (1999) [40]	1	Diagnosed at 5-6 years old during a routine check-up	Japan	Japanese	21	Unknown	Primigravidarum	Not specified	Prior to pregnancy	6	Menorrhagia	Not specified	Forceps-assisted delivery	-	80,000	Not specified	Not specified	Not specified	Not specified	No	No	Prolonged fetal bradycardia	780	MHA (-)	Uneventful	Uneventful
Scurlock et al. (2005) [41]	1	Twins	United States	Not specified	28	Yes	Primigravidarum	Not specified	Not known	Not known	No bleeding tendency	Not specified	SVD	-	Not specified	Not specified	Not specified	Not specified	Not specified	No	No	No	Not known	Twin 1: MHA (+). Twin 2: MHA (+)	Twin 1: Uneventful. Twin 2: Uneventful	Uneventful
Filanovsky et al. (2009) [42]	1	-	Israel	Not specified	24	Unknown	Primigravidarum	Routine antenatal blood test	During the current pregnancy	24	No bleeding tendency	Not specified	Not specified	Not specified	21,000	Not specified	Not specified	Not specified	Not specified	Not specified	No	Not known	Not known	Not known	Not known	Not known
Bizzaro et al. (1999) [4]	33 (4 twin births)	Data were obtained from one large family	United States of America	Italian	21-83	Yes	Not specified	Not specified	Prior pregnancy	Not specified	No bleeding tendency	Not specified	30 SVD (4 twins) and 3 LSCS	Not specified	87,000	Not specified	Not specified	Not specified	Not specified	No	No	No	Not specified	Uneventful	20 affected with MHA out of 37, with a platelet range of 48-115. Another 7 cases where the father had the anomaly, they were delivered vaginally without complications	Uneventful

Anaesthesia concerns

It is reassuring that all patients who underwent neuraxial anaesthesia in our literature search did not experience epidural-spinal haematoma. One of the case series performed neuraxial anaesthesia for seven patients with MHA who underwent vaginal deliveries and caesarean sections [10]. In our search, only 9.7% had postpartum haemorrhage, and none of them experienced spinal haematoma. According to the review of the documented literature, which spans over 54 years, technology in diagnostics and care in general has improved for obstetric patients, and the rate of postpartum haemorrhage is likely lower than the finding in this current case study.

There is no consensus for an absolute lower platelet count limit that grants safety for neuraxial anaesthesia. "The literature is insufficient to assess whether a routine platelet count can predict anaesthesia-related complications in uncomplicated parturients" [43]. Within the guideline, 77% of anaesthetists strongly agreed and 21.3% agreed that ordering platelets should be individualised and based on patient history, physical examination, and clinical signs [43]. This is also shown in our literature search, showing that mothers with underlying MHA but otherwise an uncomplicated pregnancy usually have an uneventful pregnancy and delivery. However, mothers with MHA with an added issue of placenta previa, HELLP syndrome, or preeclampsia, for example, have a tendency to experience postpartum haemorrhage (Figure 2). A thromboelastography (TEG) could also be a valuable analysis of MHA, which may guide clinicians in assessing haemostasis. A case report that analysed the TEG of preterm twins showed normal or slightly reduced platelet function despite low platelet counts (12-28 × 103 per mm^3^) [11]. The use of TEG is, however, discretionary as literature is scarce with respect to patients with MHA. To properly understand the nature of platelets in this regard requires further research.

Obstetrics concerns

Prophylactic platelet transfusion prior to delivery to reduce the risk of postpartum haemorrhage is discretionary. There were isolated postpartum haemorrhage cases, such as uterine atony and uterine inversion, which resolved without platelet transfusion [10,27,34]. The majority of patients underwent vaginal delivery, including instrument-assisted delivery, and most had uneventful outcomes. However, instrumental delivery remains a relative contraindication for patients with thrombocytopenia and should be used with caution to prevent complications such as cephalohematoma or intraventricular haemorrhage.

MHA is a rare cause of thrombocytopenia in pregnancy. It is often detected with a complete blood count, followed by a PBS due to its availability, cost-effectiveness, and interpretability, but it carries a risk of misdiagnosis as ITP [44,45]. We found 14 mothers who were misdiagnosed with ITP and were unresponsive to steroids, intravenous immunoglobulins, and splenectomy [13,15,28,29]. None of the literature reported the use of intra-abdominal drains to detect intra-abdominal bleeding and therefore may not be useful in cases of MHA. A multidisciplinary approach between the obstetrics, anaesthesia, paediatrics, and haematology teams should be carried out to plan for the anticipated delivery. Although PBS as a form of diagnosis is widely accepted, its sensitivity and specificity have never been described or observed. Understanding this may potentially reduce the misdiagnosis of ITP and reduce unnecessary surgery and expensive medication.

Paediatric concerns

Amongst the 103 infants delivered, 85.4% (n = 88) had no associated complications at birth, and 7.8% (n = 8) of infants had complications at birth. These complications were intrauterine fetal demise, bilateral temporal cephalohematoma, intracranial cyst, and ambiguous genitalia. There were two cases of intrauterine fetal demise from two separate mothers, where one was reported to have uteroplacental insufficiency as the cause, and the second case did not discuss the cause. Only one case of spontaneous bilateral temporal cephalohematoma was reported, and this baby was delivered via caesarean section. There were no further complications. In the peripartum period, 95.3% (n = 41) of infants with MHA reported no bleeding tendencies, even in infants who had instrument-assisted delivery or circumcision. This can raise an argument that, in spite of the low platelet count, the risk of bleeding is minimal. It was theorised that the absence of bleeding may be explained by the normality of platelet function guaranteed by a total mass of giant platelets equivalent to that of a normal subject [4]. Most studies suggest that individuals with MHA are asymptomatic, tests of platelet function and aggregation are typically normal, and platelet defects responsible for excessive bleeding have not been defined [2].

Limitations

Some mothers have multiple pregnancies, which may skew the data presentation. Not all data were captured in each case, and most of the articles were of low-quality evidence, which may not be ideal for describing the risks and best practices. With the interest in treating this population of patients, a global multicentre data-capturing method should be employed to further understand the clinical implications of MHA and how to approach MHA in pregnancy.

## Conclusions

MHA is a rare disease that needs a multidisciplinary approach to plan for safe delivery in obstetric patients. The literature suggests that mothers with MHA generally have an uneventful delivery via all methods. Children who were delivered did not have any complications pertaining to MHA. In terms of anaesthetics, all types of anaesthetic methods seem to be safe without direct complications.
